# Investigation of Some Clinicopathologic Alterations in Cats Infected With *Mycoplasma haemofelis* and *Candidatus Mycoplasma haemominutum* in Mashhad, Iran: An Observational Cross‐Sectional Study

**DOI:** 10.1002/vms3.70987

**Published:** 2026-05-04

**Authors:** Esmaeel Shahtahmasbi, Elham Valavi, Gholam Reza Razmi, Mahdieh Zaeemi

**Affiliations:** ^1^ Department of Clinical Sciences, Faculty of Veterinary Medicine Ferdowsi University of Mashhad Mashhad Iran; ^2^ Department of Pathobiology, Faculty of Veterinary Medicine Ferdowsi University of Mashhad Mashhad Iran

**Keywords:** cat, hemotropic mycoplasmas, microscopic observation, molecular detection, prevalence

## Abstract

**Background:**

Data on feline hemotropic mycoplasmas is limited in Iran. Aim. The aim of this study was to investigate the infection rate of cats with this pathogen in Mashhad,Iran and evaluate its assosiation with cliniopathological changes.

**Materias & Methods:**

Blood samples from 100 cats presenting at veterinary clinics were analysed for *Mycoplasma haemofelis* (Mhf) and *Candidatus Mycoplasma haemominutum* (CMhm). Samples were screened for hemotropic mycoplasmas using microscopic examination and conventional polymerase chain reaction (PCR). Samples were also subjected to haematological and biochemical analysis.

**Results:**

Microscopic examination detected hemotropic mycoplasmas in 65% of samples, whereas PCR identified a 23% infection rate, with 65.21% (*n *= 15) positive for CMhm and 34.78% (*n *= 8) for Mhf. The presence of *Haemomycoplasma* DNA was significantly associated with age, gender, breed and roaming status (*p *< 0.05). Infected cats exhibited significant alterations (*p* < 0.05) in haematocrit (Hct), red blood cell count (RBC), haemoglobin (HGB), white blood cell count (WBC), neutrophil counts, serum total protein (TP), globulin, phosphorus (Pi), aspartate aminotransferase (AST) levels and the albumin/globulin ratio (AGR).

**Conclusions:**

Our study highlights a high prevalence of feline hemoplasmosis in northeastern Iran, with CMhm as the predominant species. Key risk factors included male gender, adult, outdoor access and domestic short hair (DSH) breed. Although infected cats showed consistent clinicopathologic changes, no differences were observed between Mhf and CMhm infections.

## Highlights

- 23% Haemomycoplasma infection in Mashhad; CMhm dominant (66.22%)
- Low microscopic‐PCR agreement; PCR is more reliable for feline haemplasmas
- Infection associated with age, gender, outdoor access, and some clinical parameters

## Graphical abstract

This study detected *Mycoplasma haemofelis* (34.78%) and *Candidatus M. haemominutum* (65.21%) in 23% of Iranian cats via PCR. Infection correlated with age, gender and outdoor access (*p < 0.05), causing significant hematologic/biochemical changes. No inter‐species differences in clinical presentation emerged between Mhf and CMhm.

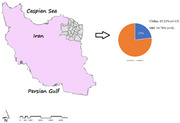

## 1 Introduction

Feline hemotropic mycoplasmosis is a disease in cats that ranges from acute to chronic, characterized by the destruction of red blood cells. The disease is caused by vector‐borne bacterial pathogens that reside within the red blood cells. These gram‐negative bacteria are fastidious and difficult to culture in a laboratory setting. Various species of hemotropic mycoplasma have been identified in cats, including *Mycoplasma haemofelis* (Mhf), *Candidatus Mycoplasma haemominutum* (CMhm) and *Candidatus* Mycoplasma turicensis (CMt) (Yasmin et al. 2022).

The disease caused by these species manifests in various clinical presentations, ranging from asymptomatic infection to acute haemolytic anaemia. Among different species, Mhf is considered the most pathogenic in cats, whereas CMhm, the most common species in cats, generally exhibits low virulence. However, the severity of the disease caused by CMhm can be increased if cats are co‐infected with other pathogens, stressed, or immunodeficient (Tasker 2010; Tasker et al. 2018).

Diagnosing feline hemoplasmosis traditionally involves microscopic examination of stained blood smears to detect organisms attached to erythrocytes. However, this method has limitations due to the potential detachment of organisms during sample storage and the difficulty in distinguishing haemoplasmas from staining artefacts or other cellular structures. Consequently, polymerase chain reaction (PCR) assays have become the preferred diagnostic tool, offering higher sensitivity and specificity by detecting low levels of hemoplasma DNA and accurately identifying different species (Tasker et al. 2018; Hoseinpoor et al. 2024; Messick and Harvey 2023).

Feline hemotropic mycoplasma infections have been reported across Asia, with varying prevalence rates in countries such as Qatar (5.9%), India (9.01%) and Saudi Arabia (13.6%) (Alho et al. 2017; Malangmei et al. 2021; Alanazi et al. 2020). Recent studies in Türkiye—a region bridging Asia and Europe—have further expanded epidemiological insights, reporting prevalence rates of 9.4%–19.3% and highlighting genetic diversity in local strains (Muz et al. 2021; Cetinkaya et al. 2016; Ceylan, Culha, et al. 2024; Ceylan, Ma, et al. 2024).

Although Iranian studies report wide‐ranging hemotropic mycoplasmas prevalence in cats (11.67%–53% by microscopy vs. 0%–25.96% by PCR) (Hoseinpoor et al. 2024; Vahedi et al. 2014; Hooshyar et al. 2016; Rassouli 2016; Ranjbar‐Bahadori et al. 2015), molecular data from Iran remain limited. The recent nationwide PCR survey (Hoseinpoor et al. 2024), while valuable for documenting 25.96% prevalence in our study region (Khorasan Razavi province), did not perform microscopic evaluation and characterize disease manifestations. Our investigation in Mashhad—a high‐referral zone for suspected cats—addresses these oversights through clinical–pathologic profiling and parallel microscopy evaluation.

## 2 Materials and Methods

### 2.1 Sample Collection

This observational cross‐sectional study included domestic cats of various ages, breeds and genders presenting at veterinary clinics in Mashhad. A random selection of cats was made for inclusion to ensure a representative sample and minimize selection bias. The selection of cats was not based on clinical signs, as infection with this pathogen can be asymptomatic in some cats, whereas others may present with visible signs such as icterus and anaemia, thereby preventing bias towards symptomatic cases. Cats were excluded if they had a recent history (within 6 months) of blood transfusions, hemoplasma treatment, antibiotic therapy, surgical procedures (except routine neutering ≥3 months prior), documented renal or hepatic insufficiency, haemorrhagic episodes or current use of immunomodulators/hematopoietic supplements to minimize potential confounders. Furthermore, all selected cats tested negative for FIV/FeLV using a commercial rapid test kit (Pet Rapid Test, Quicking Biotech Co. Ltd., China) and were regularly vaccinated with FVRCP (feline viral rhinotracheitis, calicivirus and panleukopenia) and rabies. A total of 100 jugular blood samples (each 3–4 mL) were randomly collected. The samples were collected from cats referred to private veterinary clinics in the urban area of Mashhad (36.20°N and 59.35°E) in northeastern Iran. Half of the blood volume was divided into K_3_‐EDTA tubes (FL medical, Italy) for haematological and molecular evaluation, whereas the other half was added to tubes without anticoagulant agents (WEGO, China) for biochemistry tests. The serum samples were separated immediately after clotting by centrifugation at 1800 *g* for 10 min (Jouan, C 412, France). The aliquots were stored at −80°C until analysis. The patient demographics of each animal, including gender, age and breed, were documented during the visit.

### 2.2 Staining and Microscopic Examination

To minimize hemoplasma detachment from erythrocyte surfaces and ensure accurate detection and identification, blood smears were prepared immediately after collection. These smears were air‐dried and then fixed with absolute methanol for 5 min before being stained with a 5% filtered Giemsa solution. The use of filtered Giemsa stain minimized precipitation and prevented interference in hemoplasma identification, thus ensuring accurate diagnosis. After 45 min, the slides were washed with distilled water and carefully examined for hemoplasmas under a light microscope at ×1000 (Ceylan, Culha, et al. 2024). A minimum of 100 fields was examined thoroughly before declaring a sample as negative. Hemoplasma infection was confirmed based on the observation of characteristic morphologies—including small round forms (coccobacilli, <0.8 µm), rod‐shaped forms (up to 1.5 µm) and occasional ring forms—attached to the erythrocyte membrane, as described in standard references (Tvedten 2010). All slides were independently evaluated by two experienced observers to ensure diagnostic consistency.

### 2.3 Haematological and Biochemical Analysis

Haematological analyses were performed immediately after blood collection to ensure accurate results for time‐sensitive parameters. EDTA‐anticoagulated blood was used for measuring white blood cell count (WBC) and platelet (PLT) counts, haematocrit (Hct), red blood cell (RBC) count, haemoglobin (HGB), mean corpuscular volume (MCV), mean corpuscular haemoglobin (MCH) and mean corpuscular haemoglobin concentration (MCHC). These haematological parameters were assessed using an automated veterinary haematology cell counter (Nihon Kohden MEK6450K system, Japan). Manual differential leukocyte counts were performed on blood smears, with 200 cells classified and counted to determine absolute counts for each leukocyte type. Serum biochemical analyses were conducted using commercial kits from Pars Azmoon Company (Iran) and an autoanalyzer system (Biotecnica, BT 1500, Italy). The biochemical tests included total protein (TP), albumin (ALB), total globulin (calculated by subtracting ALB from TP), total bilirubin (BIT), glucose (GLU), albumin‐to‐globulin ratio (AGR), urea, creatinine (Cr), calcium (Ca), phosphorus (Pi), alkaline phosphatase (ALP), aspartate aminotransferase (AST) and alanine aminotransferase (ALT). The accuracy of these measurements was monitored using Randox control serum (Randox, UK).

### 2.4 Molecular Detection

The genomic DNA was extracted from the animal's whole blood samples (100 µL) using DNA Isolation kits (MBST, Iran), following the manufacturer's instructions. The extracted DNA was then stored at −80°C for subsequent PCR detection of hemotropic mycoplasmas. The quality of the extracted DNA was assessed by running the samples on a 3% agarose gel.

For the molecular detection and differentiation of hemoplasmas through conventional PCR, all the extracted DNA samples were subjected to 16S rRNA amplification using forward (5′‐ACGAAAGTCTGATGGAGCAATA‐3′) and reverse (5′‐ACGCCCAATAAATCCGRATAAT‐3′) primers (Jensen et al. 2001). The size of the expected PCR product was approximately 170 bp for and 193 bp for Mhf and CMhm, respectively. PCR was performed in a 20 µL reaction volume comprising 10 µL of 2× PCR Mastermix (AMPLIQON, Denmark), 1 µM of each specified primer and 6 µL of sample DNA, and the final volume was adjusted to 20 µL by adding 2 µL of DNase‐free water. The thermal cycling conditions consisted of an initial denaturation at 94°C for 5 min, followed by 45 cycles of amplification (95°C for 2 min, 60°C for 1 min and 72°C for 30 s), and a final extension step at 72°C for 10 min, utilizing the TC3000 thermocycler (USA). Subsequently, the PCR products were separated by electrophoresis on a 3% agarose gel containing Green Viewer (10 mg/mL) (Parstous, Iran) and visualized under ultraviolet light. The positive control hemoplasma DNA samples used in our study were obtained from archived samples at the Veterinary Teaching Hospital of Ferdowsi University of Mashhad, Iran. These archived samples were collected from cats that had previously tested positive for hemoplasma infection and were stored under appropriate conditions to maintain their integrity for research purposes.

### 2.5 Statistical Analysis

Data were analysed using SPSS software, version 21. The kappa coefficient was used to evaluate agreement between molecular and microscopic methods for diagnosing hemotropic mycoplasma infection in cats. Statistical association between infection frequency and risk factors, including gender, age, breed, roaming status and clinical pathological findings, was investigated using a chi‐squared test. The normality of data distribution was assessed using the Kolmogorov–Smirnov test. Due to non‐normal distribution, haematological and biochemical parameters were compared between infected and non‐infected groups and between cats infected with Mhf and CMhm, using Kruskal–Wallis and Mann–Whitney *U* tests. A *p* value of less than 0.05 was considered statistically significant.

## 3 Results

### 3.1 Microscopic Examination

**FIGURE 1:**
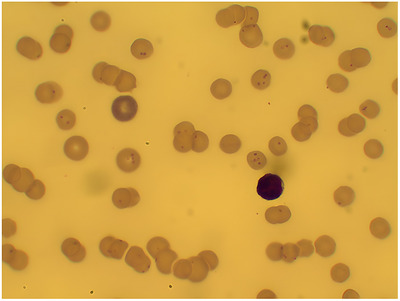
Giemsa‐stained peripheral blood smear from a cat infected with Mhf (100×) which was confirmed by PCR.

Feline hemoplasmas were identified as small, basophilic, epicellular bodies adherent to the erythrocyte surface in 65% of stained blood smear images using light microscopy. The hemoplasmas were detected as circular, single, or paired dots (Figure 1).

### 3.2 Molecular Detection

**FIGURE 2:**
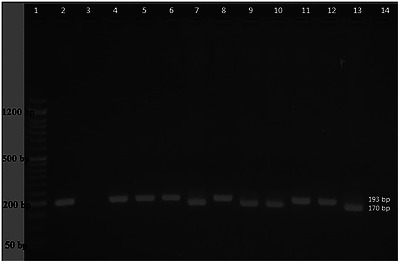
PCR products of the Mhf (170 bp) and CMhm (193 bp) 16S rRNA. Lane 1, DNA 50 bp ladder; Lanes 3 and 14, negative controls (clinically healthy cat and distilled water, respectively); Lanes 2 and 4, positive control for Mhf and CMhm, respectively, Lanes 7, 9, 10 and 13, cats that tested positive for Mhf; remaining lanes, cats that tested positive for CMhm.

Regarding molecular detection, hemoplasma DNA was detected in 23% (23/100) of tested cats (Figure 2). Of the positive cases, 8 (34.78%) were Mhf, and 15 (65.22%) were CMhm. There was a low agreement (Kappa value = 0.139) between the molecular and microscopic methods for diagnosing hemoplasma infection in cats (Table 1).

**TABLE 1:** Comparison of results of polymerase chain reaction (PCR) assay and microscopic examination for detection of hemotropic mycoplasmas in cats.

		Microscopic method		
		Positive	Negative	Total
**Molecular method**	**Positive**	19 (19%)	4 (4%)	23
	**Negative**	46 (46%)	31 (31%)	77
	**Total**	65	35	100

### 3.3 Risk Factors Associated With Feline Hemoplasmas Infection

Hemoplasma DNA presence was significantly associated with age, gender, breed and roaming status (Table 2). These risk factors were classified into two groups of sex (male and female), three age groups (<1, 1–3 and >3 years), three groups of breeds (domestic short hair [DSH], Persian and Siamese) and two groups of roaming status (stray and household cats). Cats aged 1 to 3 years were more likely to be infected with hemotropic mycoplasmas (*χ* ^2^ = 7.78, *p* = 0.02). Male cats were more susceptible to hemoplasmosis than female cats (*χ* ^2^ = 4.48, *p* = 0.034). The majority of infections were observed in DSH cats (*χ* ^2^ = 10.45, *p* = 0.005), whereas no infection was detected in Siamese cats. Regarding roaming status, stray cats (43.2%; 16/37) had higher positive rates than household cats (*χ* ^2^ = 13.6, *p* < 0.0001).

**TABLE 2:** Association of gender, age, breed, housing condition and clinicopathological variables with infection confirmed by polymerase chain reaction (PCR).

Variable (*n *= 100)	Non‐infected cats	Infected cats	*X* ^2^	*p* value
**Gender** Male (*n* = 64) Female (*n* = 36)	45 (70.3%) 32 (88.9%)	19 (29.7%) 4 (11.1%)	4.48	**0.034**
**Age**				
≤1 year (*n *= 27) 1–3 year (*n* = 50) ≥3 year (*n* = 23)	26 (96.3%) 35 (70.0%) 16 (69.6%)	1 (3.7%) 15 (30.0%) 7 (30.4%)	7.78	**0.020**
**Breed**				
Domestic short hair (*n* = 45) Persian (*n *= 50) Siamese (*n* = 5)	28 (62.20%) 44 (88.00%) 5 (100%)	17 (37.80%) 6 (12.00%) 0 (0.00%)	10.45	**0.005**
**Roaming status**				
Household (*n* = 63) Stray (*n* = 37)	55 (87.30%) 22 (59.45%)	8 (12.70%) 15 (40.54%)	10.203	**0.002**
**Hct**				
Low (*N* = 17) Normal (*n* = 83)	4 (23.53%) 73 (87.95%)	13 (76.47%) 10 (12.05%)	33.06	**0.000**
**HGB**				
Low (*n* = 14) Normal (*n* = 86)	6 (42.86%) 71 (82.56%)	8 (57.14%) 15 (17.44%)	10.71	**0.003**
**RBC**				
Low (*n* = 12) Normal (*n* = 88)	5 (41.66%) 72 (81.81%)	7 (58.33%) 16 (18.18%)	9.61	**0.005**
**MCV**				
Low (*n* = 17 Normal (*n *= 83)	15 (88.24%) 62 (74.70%)	2 (11.76%) 21 (25.30%)	1.469	0.340
**Platelet**				
Low (*n* = 88) Normal (*n* = 12)	69 (78.40%) 8 (66.67%)	19 (21.60%) 4 (33.33%)	0.822	0.424
**WBC**				
Low (*n* = 11) Normal (*n* = 78) High (*n* = 11)	6 (54.55%) 71 (91.02%) 0 (0.00%)	5 (45.45%) 7 (8.98%) 11 (100%)	48.62	**0.000**
**Neut. seg**				
Low (*n* = 7) Normal (*n* = 65) High (*n* = 28)	6 (85.71%) 55 (84.61%) 16 (57.14%)	1 (14.28%) 10 (15.38%) 12 (42.85%)	8.66	**0.013**
**Neut. band**				
Normal (*n* = 92) High (*n* = 8)	74 (80.43%) 3 (37.5%)	18 (19.56%) 5 (62.5%)	7.66	**0.015**
**Lymphocyte**				
Low (*n* = 27) Normal (*n* = 64) High (*n* = 9)	20 (74.07%) 51 (79.68%) 6 (66.66%)	7 (25.92%) 13 (20.31%) 3 (33.33%)	0.934	0.640
**Total protein**				
Normal (*n* = 78) High (*n* = 22)	67 (85.89%) 11 (45.45%)	10 (14.10%) 12 (54.54%)	**15.848**	**0.000**
**Albumin**				
Normal (*n* = 75) High (*n* = 25)	57 (77.33%) 19 (76.00%)	17 (22.66%) 6 (24.00%)	0.19	0.544
**Globulin**				
Normal (*n* = 86) High (*n* = 14)	71 (82.55%) 6 (42.85%)	15 (17.44%) 8 (57.14%)	**10.715**	**0.003**
**AGR**				
Low (*n* = 12) Normal (*n* = 72) High (*n* = 16)	4 (33.33%) 58 (80.55%) 15 (93.75%)	8 (66.66%) 14 (19.44%) 1 (6.25%)	**15.97**	**0.001**
**Urea**				
Normal (*N* = 24) High (*N* = 76)	19 (79.16%) 58 (76.31%)	5 (20.83%) 18 (23.68%)	0.084	0.506
**Creatinine**				
Normal (*n* = 64) High (*n* = 36)	51 (79.68%) 26 (72.22%)	13 (20.31%) 10 (27.77%)	0.725	0.461
**Glucose**				
Low (*n* = 12) Normal (*n* = 52) High (*n* = 36)	8 (66.66%) 41 (78.84%) 28 (77.77%)	4 (33.33%) 11 (21.15%) 8 (22.22%)	0.836	0.658
**Total bilirubin**				
Normal (*n* = 77) High (*n* = 23)	68 (88.31%) 9 (39.13%)	9 (11.69%) 14 (60.87%)	**15.99**	**0.001**
**AST**				
Normal (*n* = 18) High (*n* = 18)	66 (90.41%) 11 (40.740%)	7 (9.59%) 16 (59.26%)	**27.45**	**0.001**
**ALT**				
Normal (*n* = 85) High (*n* = 15)	67 (78.82%) 10 (66.66%)	18 (21.17%) 5 (33.33%)	1.064	0.326
**ALP**				
Normal (*n* = 61) High (*n* = 39)	46 (75.41%) 31 (79.48%)	15 (24.59%) 8 (20.51%)	0.223	0.637
**Calcium**				
Normal (*n* = 87) High (*n* = 13)	68 (78.16%) 9 (69.23%)	19 (21.83%) 4 (30.76%)	0.509	0.489
**Phosphorous**				
Low (*n* = 24) Normal (*n* = 68) High (*n* = 8)	23 (95.83%) 54 (79.41%) 0 (0.00%)	1 (4.16%) 14 (20.58%) 8 (100%)	**31.81**	**0.002**

Abbreviations: AGR, albumin‐to‐globulin ratio; ALP, alkaline phosphatase; ALT, alanine aminotransferase; AST, aspartate aminotransferase; DSH, domestic short hair; Hct, haematocrit; HGB, haemoglobin; MCV, mean corpuscular volume; PLT, platelet; RBC, red blood cell count; TP, total protein; WBC, white blood cell count.

Bold values indicate statistical significance (*p* < 0.05).

### 3.4 Haematological and Biochemical Findings

A significant association (*p* < 0.05) was observed between hemoplasma infection and various laboratory findings. The RBC count, HGB levels and Hct were significantly lower in infected cats (*p* < 0.05). Conversely, total WBC, segmented neutrophil and band cell counts were significantly elevated in infected cats compared to the non‐infected group (*p* < 0.05). Infected cats also demonstrated significantly higher levels of serum TP, total globulin, BIT, Pi and AST activity while exhibiting a significant decrease in the AGR (*p* < 0.05). Further examination of clinical signs revealed that 47.82% (11/23) of infected cats presented with icterus and anaemia. When comparing the two infected groups, no significant differences were noted in the haematological and biochemical parameters. Additional information is available in Table 3.

**TABLE 3:** Comparison of haematological and some serum biochemical parameters (Median, 25th, and 75th percentiles) between infected and noninfected cats with hemotropic mycoplasmas.* indicates a significant difference between infected and non‐infected groups. Unlike superscript letters indicate significant difference between different groups

Parameters	Groups			
	Non‐infected (*n* = 77)	Infected (*n* = 23)		
		Mhf (*n* = 8)	CMhm (*n* = 15)	Total (*n* = 23)
**Hct (%)**	32 (28–36)^a^	23 (19–37)^b^	23 (18–32)^b^	23 (19–32)*
**RBC (×106/µL)**	8.41 (7.36–9.19)^a^	6.20 (4.17–9.23)^b^	5.49 (4.56–7.20)^b^	5.73 (4.59–7.29)*
**HB (g/dL)**	11.80 (10.30–13.40)^a^	8.80 (7.00–13.45)^b^	9.35 (6.82–10.52)^b^	8.80 (6.90–10.60)*
**WBC (×10^3^/µL)**	8.55 (7.47–11.85)^b^	16.85 (6.80–32.42)^ab^	18.400 (6.60–37.10)^a^	16.60 (5.30–28.00)*
**Seg. Neutrophil (×10^3^/µL)**	5.14 (2.98–9.44)^b^	11.40 (3.84–27.65)^ab^	13.77 (5.21–23.00)^a^	12.45 (3.94–3.17)*
**Band. Neutrophil (×10^3^/µL)**	0.00 (0.00–0.00)^b^	0.16 (0.00–0.40)^a^	0.16 (0.00–1.21)^a^	0.16 (0.00–0.85)*
**Total Protein (g/dL)**	64^a^ (60–68)	78 (62–98)^ab^	88 (70–97)^b^	93 (62–100)*
**Globulin (g/dL)**	35^a^ (30–38)	50 (28–68)^ab^	53 (40–65)^b^	54 (32–72)*
**AGR**	0.84^a^ (0.78–1.06)	0.56 (0.44‐1.25)^ab^	0.62 (0.46–0.77)^b^	0.59 (0.35–0.90)*
**Total bilirubin (µmol/L)**	1.03 (0.34–1.37)^a^	4.79 (4.62–23.6)^b^	8.21 (4.28–40.53)^b^	5.47 (4.62–30.78)*
**AST (IU/L)**	28 (23–44)^a^	37 (23–105)^ab^	69 (37–107)^b^	48 (25–118)*
**Phosphorous (mmol/L)**	1.84 (1.25–2.08)^a^	3 (1.54–3.32)^ab^	2.19 (1.93–2.93)^b^	2.1 (1.4–3.0)*

*Note*: Unlike letters show significant differences between non‐infected cats and those infected with *Mycoplasma haemofelis* (Mhf) and *Candidatus* Mycoplasma haemominutum (CMhm).

Abbreviations: AGR, albumin‐to‐globulin ratio; AST, aspartate aminotransferase; Hct, haematocrit; RBC, red blood cell count; TP, total protein; WBC, white blood cell count.

## 4 Discussion

The significant discrepancy between microscopic (65% positivity) and PCR (23% positivity) results can be attributed to a combination of factors. First, microscopy has inherent limitations in specificity; stain precipitates, Howell–Jolly bodies or other erythrocytic artefacts can be misinterpreted as hemoplasma organisms, potentially leading to an overestimation of prevalence (Tasker and Lappin 2002; Martínez‐Díaz et al. 2013). Additionally, some microscopy‐positive cases could represent co‐infections with other hemotropic pathogens (e.g., *Bartonella* spp. and *Babesia* spp.).

Conversely, our PCR assay was designed to detect only the two most common species, CMhm and Mhf. Therefore, it may have failed to detect infections caused by other species, such as CMt, which has been reported in this region (Ghazisaeedi et al. 2014). This limitation in the target scope likely contributed to an underestimation of the true infection rate. Thus, we will emphasize that future studies utilizing broader‐range molecular assays are necessary to fully resolve this diagnostic discrepancy and obtain a more accurate epidemiological picture.

The infection rate of hemotropic mycoplasma in our study (23%) was substantially higher than rates reported in neighbouring countries (5.9%–13.6%) (Alho et al. 2017; Malangmei et al. 2021; Alanazi et al. 2020). Notably, our findings align with the highest reported infection rate in Iran by Hoseinpoor et al. (2024) in Khorasan Razavi Province (25.96%), whereas other regions like Kerman Province showed no detectable infection (Hoseinpoor et al. 2024). This striking geographical disparity suggests that this region may represent an epidemiological hotspot for feline hemotropic mycoplasmosis. This geographical clustering can be related to high urban stray cat density or different climate conditions, which require further investigation in future studies.

Like previous research studies (Díaz‐Regañón et al. 2018; Bergmann et al. 2017; Assarasakorn et al. 2012; Jenkins et al. 2013; Sacristán et al. 2019), CMhm appeared to be the most prevalent species in our sample set. The widespread presence of CMhm in cats could potentially be attributed to its ability to cause latent infections that do not significantly impact the cat's general health or hinder its socialization with other cats (Rosenqvist et al. 2016). Additionally, CMhm is known to establish persistent infections for extended periods compared to other feline hemoplasmas (Barker and Tasker 2013). In contrast, Mhf appeared more frequently than CMhm in Canada and an Iranian investigation (Kamrani et al. 2008; Ghazisaeedi et al. 2014), indicating regional differences in the distribution of these hemoplasma species.

The higher prevalence of hemotropic mycoplasmas in older cats (>1 year) observed in this study aligns with prior findings (Korman et al. 2012), potentially reflecting cumulative exposure or age‐related immune decline (Willi et al. 2006; Bauer et al. 2008). However, conflicting evidence exists, with some studies reporting no correlation or even an inverse relationship between age and infection risk (Hooshyar et al. 2016; Sykes et al. 2008; Torkan et al. 2013), underscoring the need for further investigation into age‐dependent susceptibility.

The increased infection rate in male cats may stem from behavioural factors, such as aggressive interactions, which facilitate transmission. Although this supports the consensus of male predisposition (Sykes et al. 2008; Ghazisaeedi et al. 2014), other studies found no significant gender‐based differences (Hooshyar et al. 2016; Torkan et al. 2013).

Consistently, outdoor access was another predisposing risk factor for hemoplasma infection in our study. Stray cats, in particular, had a greater likelihood of being exposed to the infection compared to household cats (Díaz‐Regañón et al. 2018; Spada et al. 2014). Although purebred or pedigree cats have been reported to be less susceptible to hemoplasma infection, 6 out of 23 infected cats (26.08%) in our study were Persian cats. However, no infection was detected in Siamese cats, likely due to the small sample size of this breed in our study. Yasmin et al. (2022) hypothesized that the lower risk observed in purebred cats is not necessarily associated with their breed, but rather with their typically controlled and confined home environment compared to DSH breeds.

Although our study identified significant associations between hemoplasma infection and certain haematological/biochemical alterations, these findings must be interpreted cautiously as they are non‐specific and may overlap with other conditions, including chronic inflammatory diseases or hepatobiliary disorders. Although our exclusion criteria helped minimize confounding factors, the potential influence of undetected comorbidities cannot be entirely ruled out.

Of the infected cats, 12 (56.52%) developed anaemia, which we categorized by Hct levels (Tvedten 2010) into: mild anaemia (Hct 20%–26%): 7 cases (53.8% of anemic cats); moderate anaemia (Hct 14%–19%): 3 cases (23.1%) and severe anaemia (Hct 10%–13%): 3 cases (23.1%). Notably, no cases of very severe anaemia (Hct <10%) were observed. Our findings align with reports of hemoplasma‐associated anaemia (Jensen et al. 2001; Torkan et al. 2013), though discrepancies exist in the literature (Martínez‐Díaz et al. 2013; Korman et al. 2012). This variability may reflect differences in bacterial load or strain virulence, host immune status or infection stage and geographic factors in our study population.

Although leukocytosis (47.83%) and neutrophilia (52.17%) were observed in a higher number of the infected cats in this research, there were infected cases with normal leukograms or even leukopenia and neutropenia. Differential leukocyte counts can vary in cases of hemoplasmosis (Tasker 2010; Duin et al. 2009). Although experimental Mhf infection induces inflammatory leukogram changes (leukopenia and monocytosis) (Baumann et al. 2015), our naturally infected cats showed normal values, suggesting chronic/subclinical presentations may lack this acute phase response. Hemoplasmosis may be accompanied by several modifications in serum biochemistry markers. Hyperproteinemia, hyperglobulinemia, and a remarkable reduction of AGR may suggest B cell activation and an inflammatory response during disease progression. However, upregulation of acute‐phase proteins and dehydration could also contribute to hyperproteinemia, as previously reported (Korman et al. 2012; Duin et al. 2009; Vilhena et al. 2018).

BIT levels were significantly elevated in infected cats compared to non‐infected controls (*p *< 0.01). Although hyperbilirubinemia can be associated with severe haemolytic anaemia and liver damage, the concurrent manifestation of icterus and anaemia in 47.82% of infected cases, together with markedly increased BIT (up to 30.78 µmol/L), elevated AST activity and normal ALT values, provides compelling evidence that the hyperbilirubinemia results primarily from haemolysis rather than hepatic dysfunction.

Among the observed biochemical alterations, elevated serum Pi concentrations were detected. This finding is consistent with previous reports of hemoplasma‐associated hyperphosphatemia (İder et al. 2022; Ider et al. 2024). As Pi is primarily an intracellular cation, its release into circulation likely results from intravascular haemolysis of infected erythrocytes.

Although Mhf is generally considered more pathogenic than CMhm, our study revealed no significant differences in clinico‐pathological findings between the two species. This apparent discrepancy may be attributed to several confounding factors, such as comorbidities, diagnostic limitations, and disease phase. As this study involved randomly sampled cats, concurrent diseases could have masked or altered disease manifestations. Conventional PCR detects the presence of infection but cannot quantify bacterial load. Quantitative PCR (qPCR) would better correlate pathogen burden with clinical severity, as low‐level infections may remain subclinical. Variations in infection stages (acute, subacute and chronic) might have influenced outcomes, as host responses and symptom severity often differ by phase.

## 5 Conclusions

Our study reveals important insights into feline hemoplasmosis in northeastern Iran, demonstrating a relatively high prevalence with CMhm as the dominant species. The striking discordance between microscopy and PCR confirmation underscores microscopy's limitations due to false positives, strongly advocating for PCR‐based surveillance, particularly in asymptomatic carriers. We identified male sex, outdoor access and DSH breed as significant risk factors. Although some hematologic and biochemical alterations were consistently observed across infected cats, no inter‐species differences in clinical presentation emerged between Mhf and CMhm.

### 5.1 Our Study's Limitations

1. Uncontrolled variables: These cats were randomly selected with no limitation for clinical and nutritional status 1.
2. Undetermined disease phase: The clinical stage of infection (acute, chronic, or subclinical) was not characterized, which may influence haematological and biochemical findings.
3. Limited sample size: While informative for preliminary analysis, a larger‐scale study would provide better insights into the pathogen's epidemiology and pathogenic mechanisms.
4. Limited diagnostic scope: The PCR assay was restricted to detecting only two species (CMhm and Mhf). Future work should include all locally prevalent species to avoid underestimation of infection prevalence.

## Author Contributions

**Gholam Reza Razmi**: methodology, investigation, resources, conceptualization, project administration, supervision. **Elham Valavi**: writing – original draft, investigation. **Esmaeel Shahtahmasbi**: methodology, investigation, writing – original draft. **Mahdieh Zaeemi**: funding acquisition, writing – original draft, writing – review and editing, validation, methodology, software, supervision, resources, investigation, conceptualization, project administration.

## Funding

This study was supported by Grant 3/45128 from the Vice President Research and Technology of Veterinary Medicine, Ferdowsi University of Mashhad, Iran.

## Ethics Statement

All animal experiments were performed in strict accordance with the guidelines approved by the Animal Ethics Committee of the Ferdowsi University of Mashhad, Iran (IR.UM.REC.1400.216).

## Consent

Informed consent was obtained from the owner of all cats involved in this study.

## Conflicts of Interest

The authors declare no conflicts of interest.

## Data Availability

The datasets generated during and/or analysed during the current study are available from the corresponding author on reasonable request.
